# 3-Carboxy­pyrazino[2,3-*f*][1,10]phenanthrolin-9-ium-2-carboxyl­ate

**DOI:** 10.1107/S1600536810007361

**Published:** 2010-03-03

**Authors:** Xiao-Ning Zhang, Feng Fu, Dong-Sheng Li, Cai-Xia Meng

**Affiliations:** a Department of Chemistry and Chemical Engineering, Shaanxi Key Laboratory of Chemical Reaction Engineering, Yan’an University, Yan’an, Shaanxi 716000, People’s Republic of China; b College of Mechanical & Material Engineering, Functional Materials Research Institute, China Three Gorges University, Yichang 443002, People’s Republic of China

## Abstract

In the title zwitterionic compound, C_16_H_8_N_4_O_4_, the dihedral angle between the carboxyl and carboxyl­ate groups is 72.14 (2)°. In the crystal, mol­ecules are linked by strong inter­molecular O—H⋯O^−^ and N^+^—H⋯O^−^ hydrogen bonds into double chains extended along [001]. These chains are additionally stabilized by π–π stacking inter­actions between the pyridine and benzene rings [centroid–centroid distance = 3.5542 (8) Å].

## Related literature

For coordination compounds of the title ligand, see: Weng *et al.* (2009 ▶).
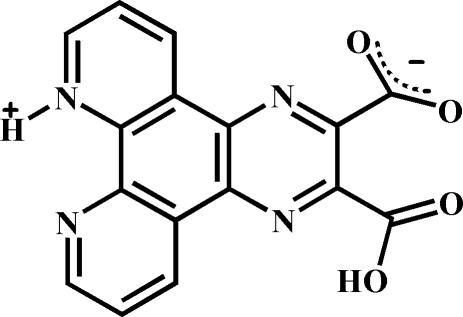


## Experimental

### 

#### Crystal data

C_16_H_8_N_4_O_4_
*M* *_r_* = 320.26
Triclinic, 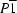

*a* = 7.302 (2) Å
*b* = 9.662 (3) Å
*c* = 10.726 (3) Å
α = 63.564 (3)°
β = 71.386 (3)°
γ = 78.283 (4)°
*V* = 640.5 (3) Å^3^
*Z* = 2
Mo *K*α radiation
μ = 0.12 mm^−1^
*T* = 293 K
0.32 × 0.21 × 0.15 mm

#### Data collection

Bruker SMART CCD diffractometer
Absorption correction: multi-scan (*SADABS*; Sheldrick, 1996 ▶) *T*_min_ = 0.969, *T*_max_ = 0.982
4652 measured reflections
2243 independent reflections
1268 reflections with *I* > 2σ(*I*)
*R*_int_ = 0.037

#### Refinement

*R*[*F* ^2^ > 2σ(*F* ^2^)] = 0.062
*wR*(*F* ^2^) = 0.177
*S* = 1.05
2243 reflections
221 parameters
1 restraint
H atoms treated by a mixture of independent and constrained refinement
Δρ_max_ = 0.38 e Å^−3^
Δρ_min_ = −0.31 e Å^−3^

### 

Data collection: *SMART* (Bruker, 1997 ▶); cell refinement: *SAINT* (Bruker, 1997 ▶); data reduction: *SAINT*; program(s) used to solve structure: *SHELXS97* (Sheldrick, 2008 ▶); program(s) used to refine structure: *SHELXL97* (Sheldrick, 2008 ▶); molecular graphics: *SHELXTL* (Sheldrick, 2008 ▶); software used to prepare material for publication: *SHELXTL*.

## Supplementary Material

Crystal structure: contains datablocks I, global. DOI: 10.1107/S1600536810007361/gk2240sup1.cif

Structure factors: contains datablocks I. DOI: 10.1107/S1600536810007361/gk2240Isup2.hkl

Additional supplementary materials:  crystallographic information; 3D view; checkCIF report

## Figures and Tables

**Table 1:** Hydrogen-bond geometry (Å, °)

*D*—H⋯*A*	*D*—H	H⋯*A*	*D*⋯*A*	*D*—H⋯*A*
O3—H3⋯O1^i^	0.82	1.82	2.638 (4)	171
N2—H2⋯O2^ii^	0.81 (2)	1.87 (3)	2.638 (4)	159 (5)

Symmetry codes: (i) 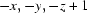; (ii) 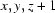.

**Fig. 1.**
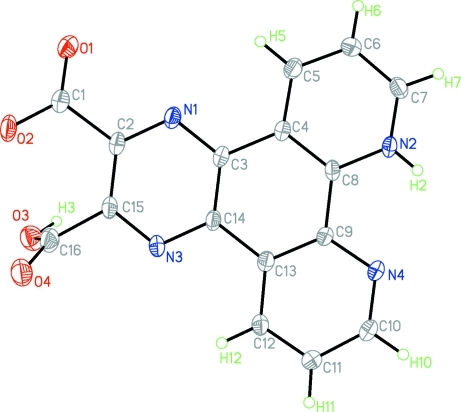
The structure of the title compound with the atom-numbering scheme showing displacement ellipsoids at the 50% probability level.

**Fig. 2.**
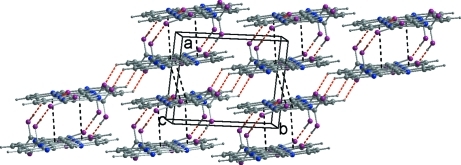
Tthree-dimensional supramolecular network formed by hydrogen bonds and π-π stacking interactions.

## supplementary crystallographic information

### Comment

[2,3-*f*]Pyrazino[1,10]phenanthroline-2,3-dicarboxylic acid (H_2_ppdb) is a multidentate O/N-donor ligand, which was rarely used up to date (Weng *et al.*, 2009). Owing to the presence of two carboxylic groups and a large conjugated π system, H_2_ppdb hoards the recognition information for the formation of interesting supramolecular structures. We attempted to synthesize a Tm^III^ complex with the H_2_ppdb ligand in hydrothermal synthesis conditions however we were unsuccessful. Instead of  crystals of the complex, single crystals of the H_2_ppdb were isolated and the structure of H_2_ppdb is reported here.

The molecular structure of title compound is shown in Fig. 1. One of the carboxyl groups is deprotonated  and the proton is transferred to the phenantroline fragment. Intermolecular hydrogen-bonding and π-π stacking interactions are the most important features of the title compound. O—H···O and N—H···O hydrogen bonds (Table 1), and  π-π stacking interactions between the pyridine rings and phenyl rings from the neighboring H_2_ppdb molecules [centroid–centroid distances = 3.5542 (8) Å] link the molecules into a double chain extending along the *c* axis. The double chains are further connected by weak C—H···O hydrogen bonds involving the carboxyl oxygen and the C—H groups adjacent to the pyridine rings and π-π stacking interactions as indicated by a center-to-center distance of 3.7402 (7) Å, forming a three-dimensional supramolecular network (Fig.2).

### Experimental

All chemicals were of analytical reagent grade, commercially available and used without further purification. A mixture of H_2_ppdb (0.1 mmol, 0.0320 g), Tm_2_O_3_(0.05 mmol, 0.0193 g) was adjusted to pH 1.5 with HNO_3 _(0.2 mol/*L*). It was then sealed in a 25 ml Teflon-lined stainless steel reactor together with water (10 ml), heated at 140 °C for 4 days, and then cooled slowly to room temperature, to furnish yellow crystals.

### Refinement

The H atom attached to N atom was located in a difference Fourier map and refined  with the N—H distance restrained to 0.82 (2) Å and *U*_iso_(H) = 1.5U_eq_(N). The other H atoms were placed in idealized positions, with C-H =  0.93 and O-H = 0.82 Å, and constrained to ride on their parent atoms with *U*_iso_(H) = 1.2Ueq(C) or *U*_iso_(H) = 1.5Ueq(O).

### Crystal data

**Table:**

C_16_H_8_N_4_O_4_	*Z* = 2
*M**_r_* = 320.26	*F*(000) = 328
Triclinic, *P*1	*D*_x_ = 1.661 Mg m^−^^3^
Hall symbol: -P 1	Mo *K*α radiation, λ = 0.71073 Å
*a* = 7.302 (2) Å	Cell parameters from 1426 reflections
*b* = 9.662 (3) Å	θ = 2.5–25.0°
*c* = 10.726 (3) Å	µ = 0.12 mm^−^^1^
α = 63.564 (3)°	*T* = 293 K
β = 71.386 (3)°	Prism, colorless
γ = 78.283 (4)°	0.32 × 0.21 × 0.15 mm
*V* = 640.5 (3) Å^3^	

### Data collection

**Table:**

Bruker SMART CCD diffractometer	2243 independent reflections
Radiation source: fine-focus sealed tube	1268 reflections with *I* > 2σ(*I*)
graphite	*R*_int_ = 0.037
phi and ω scans	θ_max_ = 25.0°, θ_min_ = 2.5°
Absorption correction: multi-scan (*SADABS*; Sheldrick, 1996)	*h* = −8→8
*T*_min_ = 0.969, *T*_max_ = 0.982	*k* = −11→11
4652 measured reflections	*l* = −12→12

### Refinement

**Table:**

Refinement on *F*^2^	Primary atom site location: structure-invariant direct methods
Least-squares matrix: full	Secondary atom site location: difference Fourier map
*R*[*F*^2^ > 2σ(*F*^2^)] = 0.062	Hydrogen site location: inferred from neighbouring sites
*wR*(*F*^2^) = 0.177	H atoms treated by a mixture of independent and constrained refinement
*S* = 1.05	*w* = 1/[σ^2^(*F*_o_^2^) + (0.0802*P*)^2^ + 0.2*P*] where *P* = (*F*_o_^2^ + 2*F*_c_^2^)/3
2243 reflections	(Δ/σ)_max_ < 0.001
221 parameters	Δρ_max_ = 0.38 e Å^−^^3^
1 restraint	Δρ_min_ = −0.31 e Å^−^^3^

### Special details

**Table:**

Geometry. All e.s.d.'s (except the e.s.d. in the dihedral angle between two l.s. planes) are estimated using the full covariance matrix. The cell e.s.d.'s are taken into account individually in the estimation of e.s.d.'s in distances, angles and torsion angles; correlations between e.s.d.'s in cell parameters are only used when they are defined by crystal symmetry. An approximate (isotropic) treatment of cell e.s.d.'s is used for estimating e.s.d.'s involving l.s. planes.
Refinement. Refinement of *F*^2^ against ALL reflections. The weighted *R*-factor *wR* and goodness of fit *S* are based on *F*^2^, conventional *R*-factors *R* are based on *F*, with *F* set to zero for negative *F*^2^. The threshold expression of *F*^2^ > σ(*F*^2^) is used only for calculating *R*-factors(gt) *etc*. and is not relevant to the choice of reflections for refinement. *R*-factors based on *F*^2^ are statistically about twice as large as those based on *F*, and *R*- factors based on ALL data will be even larger.

### Fractional atomic coordinates and isotropic or equivalent isotropic displacement parameters (Å^2^)

**Table:**

	*x*	*y*	*z*	*U*_iso_*/*U*_eq_	
O1	0.1681 (4)	−0.2077 (3)	0.4832 (3)	0.0468 (8)	
O2	0.2929 (4)	−0.0009 (3)	0.2840 (3)	0.0473 (8)	
O3	0.0845 (4)	0.3107 (4)	0.2623 (3)	0.0529 (8)	
H3	0.0010	0.2742	0.3381	0.079*	
O4	0.4003 (5)	0.3285 (4)	0.1966 (3)	0.0551 (9)	
N1	0.2349 (4)	−0.0766 (4)	0.6468 (3)	0.0324 (8)	
N3	0.2887 (5)	0.2420 (4)	0.5116 (3)	0.0374 (8)	
N2	0.2403 (5)	−0.0825 (4)	1.0943 (3)	0.0317 (8)	
N4	0.3104 (4)	0.2237 (4)	0.9626 (3)	0.0336 (8)	
C4	0.2334 (5)	−0.0835 (4)	0.8748 (4)	0.0285 (9)	
C9	0.2928 (5)	0.1575 (4)	0.8803 (4)	0.0285 (9)	
C14	0.2812 (5)	0.1585 (4)	0.6532 (4)	0.0291 (9)	
C15	0.2689 (5)	0.1654 (5)	0.4411 (4)	0.0348 (10)	
C6	0.1733 (5)	−0.3119 (4)	1.0965 (4)	0.0352 (10)	
H6	0.1440	−0.4150	1.1477	0.042*	
C8	0.2571 (5)	−0.0055 (4)	0.9502 (4)	0.0275 (9)	
C1	0.2339 (5)	−0.0776 (5)	0.4193 (4)	0.0359 (10)	
C3	0.2491 (5)	0.0009 (4)	0.7207 (4)	0.0284 (9)	
C13	0.3009 (5)	0.2401 (4)	0.7332 (4)	0.0302 (9)	
C12	0.3264 (6)	0.3985 (5)	0.6710 (4)	0.0364 (10)	
H12	0.3301	0.4574	0.5742	0.044*	
C7	0.2011 (5)	−0.2294 (5)	1.1653 (4)	0.0357 (10)	
H7	0.1920	−0.2783	1.2637	0.043*	
C10	0.3366 (6)	0.3728 (5)	0.8992 (4)	0.0392 (10)	
H10	0.3499	0.4194	0.9550	0.047*	
C5	0.1900 (5)	−0.2382 (4)	0.9509 (4)	0.0343 (9)	
H5	0.1722	−0.2920	0.9027	0.041*	
C2	0.2468 (5)	0.0059 (5)	0.5071 (4)	0.0319 (9)	
C11	0.3458 (6)	0.4662 (5)	0.7535 (4)	0.0419 (11)	
H11	0.3647	0.5712	0.7141	0.050*	
C16	0.2599 (7)	0.2711 (5)	0.2886 (5)	0.0442 (11)	
H2	0.250 (7)	−0.035 (5)	1.138 (4)	0.066*	

### Atomic displacement parameters (Å^2^)

**Table:**

	*U*^11^	*U*^22^	*U*^33^	*U*^12^	*U*^13^	*U*^23^
O1	0.062 (2)	0.0476 (19)	0.0407 (17)	−0.0151 (16)	−0.0143 (14)	−0.0215 (15)
O2	0.063 (2)	0.062 (2)	0.0262 (16)	−0.0189 (16)	−0.0099 (14)	−0.0215 (14)
O3	0.052 (2)	0.057 (2)	0.0469 (18)	−0.0093 (16)	−0.0152 (15)	−0.0159 (16)
O4	0.066 (2)	0.066 (2)	0.0367 (17)	−0.0204 (18)	−0.0114 (16)	−0.0185 (16)
N1	0.0352 (19)	0.040 (2)	0.0284 (18)	−0.0048 (15)	−0.0108 (14)	−0.0173 (15)
N3	0.051 (2)	0.040 (2)	0.0215 (17)	−0.0109 (16)	−0.0091 (15)	−0.0102 (15)
N2	0.039 (2)	0.038 (2)	0.0243 (18)	−0.0015 (16)	−0.0131 (15)	−0.0153 (15)
N4	0.042 (2)	0.036 (2)	0.0307 (17)	−0.0063 (15)	−0.0120 (15)	−0.0178 (16)
C4	0.028 (2)	0.036 (2)	0.0259 (19)	−0.0023 (17)	−0.0081 (16)	−0.0156 (17)
C9	0.032 (2)	0.032 (2)	0.026 (2)	−0.0039 (17)	−0.0074 (16)	−0.0151 (17)
C14	0.031 (2)	0.038 (2)	0.0234 (19)	−0.0035 (17)	−0.0099 (16)	−0.0140 (17)
C15	0.043 (2)	0.043 (3)	0.025 (2)	−0.006 (2)	−0.0110 (18)	−0.0165 (19)
C6	0.041 (2)	0.030 (2)	0.031 (2)	−0.0048 (18)	−0.0075 (18)	−0.0106 (18)
C8	0.028 (2)	0.035 (2)	0.0211 (19)	−0.0038 (17)	−0.0079 (15)	−0.0110 (17)
C1	0.036 (2)	0.044 (3)	0.033 (2)	−0.002 (2)	−0.0114 (19)	−0.019 (2)
C3	0.028 (2)	0.036 (2)	0.028 (2)	−0.0012 (17)	−0.0086 (16)	−0.0179 (18)
C13	0.030 (2)	0.036 (2)	0.027 (2)	−0.0042 (17)	−0.0063 (16)	−0.0151 (18)
C12	0.044 (2)	0.043 (3)	0.024 (2)	−0.012 (2)	−0.0076 (17)	−0.0122 (19)
C7	0.042 (2)	0.034 (2)	0.026 (2)	−0.0012 (19)	−0.0110 (18)	−0.0072 (18)
C10	0.050 (3)	0.040 (3)	0.039 (2)	−0.006 (2)	−0.017 (2)	−0.021 (2)
C5	0.038 (2)	0.038 (2)	0.033 (2)	−0.0047 (18)	−0.0085 (18)	−0.0193 (19)
C2	0.030 (2)	0.046 (3)	0.026 (2)	−0.0032 (18)	−0.0076 (17)	−0.0194 (19)
C11	0.056 (3)	0.038 (2)	0.036 (2)	−0.014 (2)	−0.012 (2)	−0.014 (2)
C16	0.046 (3)	0.057 (3)	0.035 (2)	−0.016 (2)	−0.010 (2)	−0.020 (2)

### Geometric parameters (Å, °)

**Table:**

O1—C1	1.244 (4)	C9—C8	1.449 (5)
O2—C1	1.270 (4)	C14—C3	1.398 (5)
O3—C16	1.341 (5)	C14—C13	1.450 (5)
O3—H3	0.8200	C15—C2	1.400 (5)
O4—C16	1.196 (5)	C15—C16	1.510 (5)
N1—C2	1.330 (4)	C6—C5	1.373 (5)
N1—C3	1.346 (4)	C6—C7	1.380 (5)
N3—C15	1.323 (5)	C6—H6	0.9300
N3—C14	1.354 (4)	C1—C2	1.520 (5)
N2—C7	1.319 (5)	C13—C12	1.397 (5)
N2—C8	1.360 (4)	C12—C11	1.365 (5)
N2—H2	0.808 (19)	C12—H12	0.9300
N4—C10	1.316 (5)	C7—H7	0.9300
N4—C9	1.347 (4)	C10—C11	1.402 (5)
C4—C5	1.393 (5)	C10—H10	0.9300
C4—C8	1.392 (5)	C5—H5	0.9300
C4—C3	1.458 (5)	C11—H11	0.9300
C9—C13	1.404 (5)		
			
C16—O3—H3	109.5	N1—C3—C14	121.7 (3)
C2—N1—C3	116.5 (3)	N1—C3—C4	118.7 (3)
C15—N3—C14	116.4 (3)	C14—C3—C4	119.6 (3)
C7—N2—C8	121.8 (3)	C12—C13—C9	117.8 (3)
C7—N2—H2	120 (3)	C12—C13—C14	123.1 (3)
C8—N2—H2	118 (3)	C9—C13—C14	119.1 (3)
C10—N4—C9	117.1 (3)	C11—C12—C13	119.4 (4)
C5—C4—C8	118.4 (3)	C11—C12—H12	120.3
C5—C4—C3	122.8 (3)	C13—C12—H12	120.3
C8—C4—C3	118.8 (3)	N2—C7—C6	121.3 (4)
N4—C9—C13	123.0 (3)	N2—C7—H7	119.3
N4—C9—C8	117.6 (3)	C6—C7—H7	119.3
C13—C9—C8	119.4 (3)	N4—C10—C11	124.5 (4)
N3—C14—C3	121.2 (3)	N4—C10—H10	117.7
N3—C14—C13	117.5 (3)	C11—C10—H10	117.7
C3—C14—C13	121.3 (3)	C6—C5—C4	120.6 (3)
N3—C15—C2	122.3 (3)	C6—C5—H5	119.7
N3—C15—C16	112.3 (3)	C4—C5—H5	119.7
C2—C15—C16	125.2 (3)	N1—C2—C15	121.7 (3)
C5—C6—C7	118.5 (4)	N1—C2—C1	118.1 (3)
C5—C6—H6	120.8	C15—C2—C1	120.1 (3)
C7—C6—H6	120.8	C12—C11—C10	118.1 (4)
N2—C8—C4	119.4 (3)	C12—C11—H11	121.0
N2—C8—C9	118.8 (3)	C10—C11—H11	121.0
C4—C8—C9	121.8 (3)	O4—C16—O3	120.5 (4)
O1—C1—O2	127.5 (4)	O4—C16—C15	121.8 (4)
O1—C1—C2	119.1 (3)	O3—C16—C15	117.4 (4)
O2—C1—C2	113.4 (4)		
			
C10—N4—C9—C13	−0.1 (5)	C8—C9—C13—C14	−2.5 (5)
C10—N4—C9—C8	−177.9 (3)	N3—C14—C13—C12	0.1 (5)
C15—N3—C14—C3	−1.6 (5)	C3—C14—C13—C12	−177.7 (3)
C15—N3—C14—C13	−179.5 (3)	N3—C14—C13—C9	179.6 (3)
C14—N3—C15—C2	−1.7 (6)	C3—C14—C13—C9	1.8 (5)
C14—N3—C15—C16	174.5 (3)	C9—C13—C12—C11	1.2 (6)
C7—N2—C8—C4	−0.4 (5)	C14—C13—C12—C11	−179.3 (3)
C7—N2—C8—C9	177.6 (3)	C8—N2—C7—C6	−0.6 (6)
C5—C4—C8—N2	1.0 (5)	C5—C6—C7—N2	0.9 (6)
C3—C4—C8—N2	179.2 (3)	C9—N4—C10—C11	0.5 (6)
C5—C4—C8—C9	−176.9 (3)	C7—C6—C5—C4	−0.2 (6)
C3—C4—C8—C9	1.3 (5)	C8—C4—C5—C6	−0.7 (5)
N4—C9—C8—N2	1.0 (5)	C3—C4—C5—C6	−178.8 (3)
C13—C9—C8—N2	−176.9 (3)	C3—N1—C2—C15	−1.2 (5)
N4—C9—C8—C4	178.8 (3)	C3—N1—C2—C1	179.3 (3)
C13—C9—C8—C4	1.0 (5)	N3—C15—C2—N1	3.3 (6)
C2—N1—C3—C14	−2.1 (5)	C16—C15—C2—N1	−172.4 (4)
C2—N1—C3—C4	178.9 (3)	N3—C15—C2—C1	−177.2 (3)
N3—C14—C3—N1	3.7 (6)	C16—C15—C2—C1	7.1 (6)
C13—C14—C3—N1	−178.5 (3)	O1—C1—C2—N1	16.9 (5)
N3—C14—C3—C4	−177.3 (3)	O2—C1—C2—N1	−163.8 (3)
C13—C14—C3—C4	0.5 (5)	O1—C1—C2—C15	−162.6 (4)
C5—C4—C3—N1	−4.9 (5)	O2—C1—C2—C15	16.8 (5)
C8—C4—C3—N1	177.0 (3)	C13—C12—C11—C10	−0.9 (6)
C5—C4—C3—C14	176.1 (3)	N4—C10—C11—C12	0.0 (6)
C8—C4—C3—C14	−2.0 (5)	N3—C15—C16—O4	73.0 (5)
N4—C9—C13—C12	−0.7 (6)	C2—C15—C16—O4	−110.9 (5)
C8—C9—C13—C12	177.0 (3)	N3—C15—C16—O3	−100.7 (4)
N4—C9—C13—C14	179.8 (3)	C2—C15—C16—O3	75.3 (5)

### Hydrogen-bond geometry (Å, °)

**Table:**

*D*—H···*A*	*D*—H	H···*A*	*D*···*A*	*D*—H···*A*
O3—H3···O1^i^	0.82	1.82	2.638 (4)	171
N2—H2···O2^ii^	0.81 (2)	1.87 (3)	2.638 (4)	159 (5)

Symmetry codes: (i) −*x*, −*y*, −*z*+1; (ii) *x*, *y*, *z*+1.
